# Cancer Stem Cells: Plasticity Works against Therapy

**Published:** 2015

**Authors:** T. V. Vinogradova, I. P. Chernov, G. S. Monastyrskaya, L. G. Kondratyeva, E. D. Sverdlov

**Affiliations:** Shemyakin-Ovchinnikov Institute of Bioorganic Chemistry, Russian Academy of Sciences

**Keywords:** hierarchical structure of the tumor, clonal evolution, cancer, cancer stem cells, stem cells

## Abstract

Great successes in identification and deciphering of mechanisms of the adult stem cells regulation have given rise to the idea that stem cells can also function in tumors as central elements of their development, starting from the initial stage and continuing until metastasis. Such cells were called cancer stem cells (CSCs). Over the course of intense discussion, the CSCs hypothesis gradually began to be perceived as an obvious fact. Recently, the existence of CSCs has been indeed confirmed in a number of works. However, when are CSCs universal prerequisites of tumors and to what extent their role is essential for tumor evolution remains an issue far from resolved. Likewise, the problem of potential use of CSCs as therapeutic targets remains unsolved. The present review attempts to analyze the issue of cancer stem cells and the potential of targeting them in tumor therapy.

## INTRODUCTION

Major advances in the identification and decoding of the mechanisms of adult stem cells regulation have given rise to the idea that stem cells can also function in tumors, acting as a “driving force” behind their development, all the way from the initial stage to metastasis. Such cells were called cancer stem cells (CSCs). The importance of the issue hah led to numerous publications devoted to these hypothetical central players in the development of cancer. With time, the hypothesis has begun to be perceived as a fact, as a kind of dogma that is accepted without question [1]. Indeed, the existence of CSCs has been recently confirmed in a number of studies. However, when their role is crucial for tumor evolution and when their presence is a prerequisite for malignant tumor evolution is far from resolved. Likewise, the issue of the potential use of CSCs as targets for tumor therapy remains unsolved.

The present review attempts to analyze the issue of cancer stem cells and the feasibility of targeting them in tumor therapy. We will not address a number of pertinent issues related to the regulation of the molecular mechanisms of oncogenesis. Neither will we discuss the very important issue of resistance to therapy, which has been covered in recent reviews [2-11].

Most tumors are of monoclonal origin. However, by time they are detected, they consist of genetically, phenotypically, and epigenetically heterogeneous clones. Two key hypotheses have been put forward to account for this heterogeneity: cancer stem cells and the clonal (stochastic) evolution model [12-19]. Even though these two concepts share common provisions, they are fundamentally different and imply different approaches to the treatment of tumors [12]. A number of theories combining these two concepts have been developed in recent years [20, 21]. A major driving force behind this unification approach has been the data obtained by large-scale sequencing of cancer cell genomes [22]

## CLASSICAL STOCHASTIC CLONAL EVOLUTION MODEL

**Fig. 1:**
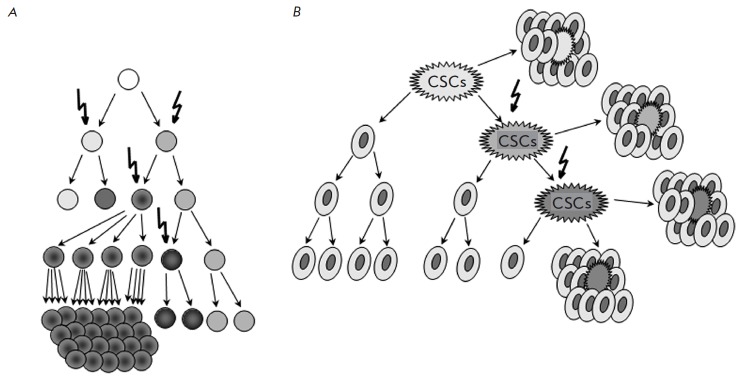
Models of heterogeneity in solid cancer cells. (A) The clonal evolution model assumes that every cell in a tumor is potentially tumor-initiating. Progression is governed by rare stochastic events operating in all cells. Cells with mutations that acquire a growth advantage will dominate over all other cells in the tumor and will originate a new clone containing cells characterized by a different phenotype and having different proliferative potentials; in a clonogenicity or tumorigenicity assay, some of these cells would have a low probability of exhibiting this potential (modified from: https://egtheory.wordpress.com/2014/10/25/stochastic-cancer). (B) The cancer stem cell model states that a particular subset of tumor cells with stem cell-like properties, called “cancer stem cell” (CSC), drives tumor initiation, progression, and recurrence. CSCs are able to self-renew indefinitely and to differentiate, leading to the production of all cell types that make up the rest of the tumor. In clonogenic assays, CSCs have the potential to proliferate extensively and can form new tumors on transplantation (modified from: http://www.hindawi.com/journals/jo/2008/492643/fig1). Stochastic mutations are shown by broken arrows

We will start with the classical model that considers evolution of cancer in terms of Darwinian evolution, where cells more or less adapted to survival in a tumor are competing with each other [1, 12, 22-25]. Chronologically, the first model to consider is a stochastic evolution model, which is represented schematically in *Fig. 1A*.

Intratumoral heterogeneity has been traditionally assessed using a stochastic model proposed by Peter Nowell in 1976 [26] (e.g., recent reviews [23, 27, 28]). Nowell relied on data on chromosomal heterogeneity in evolving tumors available at the time. His central idea is a concept of a clone defined as a group of cells derived from the same progenitor cell. Clonal expansion occurs when cells with advantage in fitness (e.g., growth rate) are selected in the course of evolution. Therefore, it implies that genetic and epigenetic changes arising from mutations in cells can be selected and produce clones with different numbers of cells. Since many different clones are selected in the course of tumor evolution, the tumor becomes polyclonal, even though all cells and clones are derived from a single progenitor (*Fig. 1A*). This type of polyclonal structure has been confirmed by the sequencing of the genomes of various types of tumors [28].

These cells are genetically and epigenetically different, creating a huge heterogeneity within the tumor [29]. In terms of further evolution, they can also have different functionality, with some of them being more aggressive [30] and, ultimately, leading to metastasis. It is important to stress that mutations occur stochastically; there are no cells or regions in the genome which are favorable to mutations. It has been assumed that cancer cells have a mutator phenotype [9, 31, 32], which accounts for the general non-selective increase in the mutation rate in “cancerous” genomes compared to normal ones.

It is important to remember that all these processes occur in a particular environment, a niche that can be referred to as a ‘within-a-body’ ecosystem [14]. This ecosystem has a major impact on close selection [33]. It is different in each individual, and this factor, apparently, largely defines the unpredictability of individual tumor development in each patient.

It should also be noted that each malignant tumor is characterized by a large variety of mutations that are different in each individual and can have a different impact on the development of the tumor and emergence of resistance to therapy [26]. The clonal evolution model and CSCs model are not mutually exclusive, since the evolution of CSCs most likely also follows the laws of clonal evolution [27].

Already back in 1976, Nowell offered a very interesting insight into the individual differences in tumors: “One may ultimately have to consider each advanced malignancy as an individual therapeutic problem after as many cells as possible have been eliminated through the nonspecific modalities of surgery, radiation, and chemotherapy. Then, perhaps, immunotherapy becomes a leading candidate for the easiest means of destroying the remainder of the neoplastic clone [26].”

Recently, an interesting stochastic clonal model has been proposed in which all cells are regarded as phenotypically similar to stem cells (stemness phenotype model, SPM) [34]. The term “stemness” [35, 36] has been gaining popularity in recent years and includes all the properties attributed to stem cells; in particular, the ability to self-renew and differentiate. There are attempts to apply this term to CSCs in a more general sense, referring to the ability to maintain and regulate the state of a stem cell [21]. SPM cells possess the properties of stem cells to a greater or lesser degree; upon implantation, they can initiate the development of a tumor; however, in principle, any cancer cell can be tumorigenic. Therefore, according to this model all tumor cells must be considered as targets for elimination in order to defeat the cancer.

There are other models, closely resembling the SPM (for review, see [37]). We will return to this model in the discussion of CSCs-related issues.

It should be noted that the use of any model must take into account that a tumor is a stochastic complex dynamic structure with unpredictable behavior, or, in other words, trajectory of development [38].

## DO CSCS EXIST? THE DEFINITION IS ALREADY A CHALLENGE

The general concept of CSCs is represented in *Fig. 1B*. First of all, we should define CSCs and their distinctive characteristics. CSCs-related issues were discussed in recent reviews [6, 21, 35, 39-43], and in other publications cited in this paper.

The obvious role of stem cells during embryogenesis [39, 44-47] and the hypothesis that a normal stem cell can become one in which the process of malignant transformation begins [39, 48, 49] were logically extended to tumors and has become the foundation of the CSCs model [50, 51]. A large number of articles have been devoted to CSCs. Sometimes, CSCs are referred to as tumor-initiating cells (TIC) or tumor-propagating cells (TPC) [1, 43]. We will use the term CSCs.

Eventually, both the hypothesis of CSCs and their existence began to be perceived as self-evident, as a kind of dogma which was accepted without question [1]. Nevertheless, the existence of CSCs and the exact criteria that distinguish CSCs from other cancer cells remain unresolved and intensely debated issues.

The concept of cancer stem cells was first proposed in the middle of the year 1990 (for review, see [21, 52-56]) and has since become the subject of intense discussions and clarifications, both in terms of its substance and, which is very important, in terms of nomenclature. This concept is based on the trajectory which a normal stem cell follows during differentiation. Initially, it was believed that mutations leading to cancer occur in a normal stem cell and result in its transformation into a cancer stem cell. The ability of a normal stem cell to self-renew and differentiate was automatically attributed to putative cancer stem cells. In this case, CSCs must always produce new cells: cancer stem cells and more differentiated progenies that are capable of only a limited number of divisions. Mutations accumulate during CSCs division. More differentiated cells have a limited ability to mutate, and their genome reflects the state of the cancer stem cell from which they originate. Clones are produced. Although it is generally believed that a cancerous cell is derived from a normal stem cell, during the development of a tumor CSCs can arise from various tumor cells, including differentiated ones, by the process of dedifferentiation (see below).

In 2011, a Workshop on CSCs developed key recommendations on definitions set forth in [43]. According to these recommendations, the term CSCs refers to neoplastic cells that can propagate or maintain an invasive solid tumor or leukemia over an indefinite or prolonged period of time.

This definition is apparently the result of major efforts to reach a consensus, since there are many other definitions in use that include, for example, such an important property as self-renewal

Here are some definitions from the latest reviews published in the most prestigious journals:

*Nature Review Cancer*, 2012 [57]: “We have chosen to define CSCs as the cells within a malignant clonal population that can propagate the cancer ... This definition assumes that not all of the cells within a population of malignant cells have this property … This definition also implies that CSCs are responsible for generating all of the cells within the malignant population that lack cancer-propagating ability (as well as those cells that perpetuate it). It also implies that the choice of these alternative fates by CSCs is embedded in an intrinsically established intracellular molecular response network that is likely to be related to the tissue from which the CSCs originate and that the loss of cancer-propagating ability is not readily reversible *in vivo.*”

*Cell Cycle*, 2013 [58]: “[The concept of CSCs states] that as in the normal somatic stem cells (SSC) … a small population of cells, the cancer stem cells (CSC), would reproduce *ad infinitum *and generate the very diverse, limited lifespan, multilineage differentiated majority of cells in a cancer, called the derived population cells.”

*Cell Stem Cell*, 2012. [42]: “The cancer stem cell (CSC) model postulates a hierarchical organization of cells such that only a small subset is responsible for sustaining tumorigenesis and establishing the cellular heterogeneity inherent in the primary tumor. Although CSCs exhibit the stem cell properties of self-renewal and differentiation, they do not necessarily originate from the transformation of normal tissue stem cells.”

*Nature Review Drug Discovery*, 2014 [6]: “The cancer stem cell (CSC) hypothesis posits the existence of subpopulations of neoplastic cells within a tumor that exhibit an elevated ability to seed new tumors upon experimental implantation in appropriate animal hosts. Implicit in this power is the ability of such cells to divide asymmetrically, yielding daughters that remain as CSCs (the trait of self-renewal) as well as daughters that differentiate into the neoplastic cells forming the bulk of the tumor… The existence of multiple subpopulations within a tumor with distinct tumor-initiating powers is no longer a matter of speculation and hypothesis. Accordingly, the use of the term “cancer stem cell paradigm” now seems to be more appropriate… To date most CSCs are not known to differentiate into more than a single cell type – the cells composing the bulk of the tumor… The phenotypes of CSCs are complex, variable from one tumor to another … hence CSCs are often difficult to rigorously define by associating them with traits beyond their shared functional trait of tumor-initiating ability. Moreover, the existence of CSCs within tumors implies that cancer cells sharing a common genetic make-up can nevertheless exist in at least two alternative phenotypic states – CSCs and non-CSCs.”

*Cell Stem Cell*, 2014 [21]: “For many cancers, CSCs represent a distinct population that can be prospectively isolated from the remainder of the tumor cells and can be shown to have clonal long-term repopulation and self-renewal capacity—the defining features of a CSC. However, in some cancer types it has not been possible to distinguish CSCs from non-CSCs because most cells have CSC function. Such tumors seem to be homogeneous or possess a very shallow hierarchy.”

Self-renewal and long-term ability to generate more differentiated cancerous cells were added to the requirements only in 2015 [59].

It should be emphasized that according to the CSCs concept, CSCs form a *separate *population in a tumor, which differs from the bulk of the tumor in their ability to initiate new tumors when implanted, self-renew and exhibit the presence of phenotypic markers, which distinguish them from the bulk. In general, the concept of CSCs is a hierarchical model with CSCs as the source; therefore, the CSCs model is sometimes referred to as hierarchical. It is very similar to the hierarchy observed in adult stem cells, which produce a sequence of more differentiated cells.

Thus, according to the concept, the CSCs population has the following features:

1. It constitutes a small proportion of the total population of tumor cells.

2. It expresses a specific set of characteristic surface markers that distinguish it from the bulk of other cells.

3. In contrast to other tumor cell populations, it selectively retains the ability to initiate tumors

4. It supports the growth of a heterogeneous mass containing the full repertoire of partially (or fully) differentiated cancer cells capable of several differentiations or at the stage of final differentiation.

5. It forms a separate pool of cells that can be identified by biological and physico-chemical methods. (There should be at least two pools of cells in tumors: CSCs and their derivative cells that are differentiated to different extents [1].)

6. Just like regular stem cells, it displays an ability for unlimited self-renewal and differentiation in many directions [37].

7. It exhibits high resistance to standard therapy. The possible reasons for the increased resistance of putative CSCs have been detailed in recent reviews [37, 60]. They include selective expression of some members of the family of multidrug resistance transporters, increased expression of anti-apoptotic molecules, increased DNA repair capacity, activation of specific stem cell survival signals (prosurvival signaling), in particular, Notch, Hedgehog (Hh), Wnt, JAK/STAT, and others. However, this issue remains a subject of debate and more studies are required, primarily those that would confirm that the observed effects are associated with CSCs.

It is important to understand that CSCs (and cancer cells in general) are characterized by higher intratumoral heterogeneity. They form subclones in the tumor, but within a subclone each cell differs from the other in the structure of its genome, the nature of transcriptome, proteome, etc. In the course of tumor development, this heterogeneity leads to different and constantly changing combinations of molecular defects in the subclones. Since the CSCs that drive the reproduction of various subclones are different, the subclones have different malignant potentials, can exist in different intratumoral microenvironments, and have different ways of interacting with these microenvironments.

Consequently, every existing and dividing subclone must contain its own CSCs compartment with unique genomic and epigenetic characteristics and each subclone can give rise to new subclones, whose properties will be different from the original subclone. If a new cell with increased malignant potential appears in a new subclone, it will become a new CSC and will be able to produce new CSCs.

The existence of human CSCs has been confirmed experimentally by demonstrating the ability of cells obtained directly from a patient to produce a malignant derivative population when transplanted to immunodeficient mice. An inherent phenotypic difference allowing to physically separate CSCs from the bulk of cells, for example, a surface antigen, is used to demonstrate that CSCs are truly different from the bulk of tumor cells.

Identification of surface antigens characteristic of CSCs is the subject of many experimental papers and reviews, and the list of such markers is constantly growing. *Table 1* lists some of the markers and intracellular proteins that are characteristic of putative CSCs. They will be discussed below.

**Table 1:** Typical surface antigens that occur with increased frequency in putative CSCs and were targeted in clinical trials [59] (see also Ref. [105])

Surface antigen	Type of cancer, containing the antigen in CSCs	Drug used
CD20	ALL, CLL	Rituximab
CD33	CML, AML	GO
CD44	AML	mAb
CD52	5q-AML, CLL	Alemtuzumab
CD123	AML	mAb
EGFR	Colon-Ca	Cetuximab
HER2/neu	Breast/Gastric/Ovarian-Ca	Trastuzumab

Note. ALL, acute lymphoblastic leukemia; CLL, chronic lymphocytic leukemia; CML, chronic myeloid leukemia; AML, acute myeloid leukemia; mAb, monoclonal antibody; Ca, carcinoma.

The putative CSCs can be separated from the bulk of tumor cells by flow cytometry using specific surface markers [61, 62]. The isolated cells have higher tumorigenicity in case of xenotransplantation to immunocompetent animals. The essential biochemical characteristic of these cells is overexpression of cytoprotective enzymes, such as aldehyde dehydrogenase (ALDH), and pumps that remove toxic compounds from the cells; for example, ABC-transporters [61, 62].

This distinction, however, is not universal, because there are CSCs that do not possess these markers and those with non-CSCs markers [35, 63]. Thus, the markers are not stable phenotypic characteristics; they can vary from one individual to another and change at different stages of tumor development. Therefore, phenotypic data alone cannot be used as sufficient proof of the presence of a separate CSCs population. It is assumed that the mechanisms that define the specific properties of CSCs are unstable because they can be associated with changes in the epigenome, which are frequent in tumor populations [43]. There are other reasons to believe that the CSCs phenotype can be very unstable [43].

Some aforementioned CSCs characteristics, in particular asymmetric division and irreversible process of transition to a differentiated state, are either not proven or seem unlikely [1], and the irreversibility of the transition has been disproved in recent experiments (see below). The claim that CSCs represent a relatively small fraction of the general population of cells should also be treated with caution, since a fraction of CSCs can vary greatly (from 0.1 to 30%) depending on the tumor type and design of the experiment [27, 64-66].

It seems that the authors of the review [27] are right to believe that some types of cancers develop through CSCs, while others do not. It has even been suggested that this may apply to the same type of cancer in different patients.

If the concept of CSCs is true, then in practical terms this implies that all the measurements that we perform, e.g., full genome sequencing, refer to the bulk of the tumor which has already ceased active division and accumulation of mutations. And the CSCs that represent an absolute minority and continue to actively divide after the separation of the bulk of the tumor and acquire mutations which give them new functionalities are factored out. Any positive outcome in a study of the total mass should be attributed to the fact that all the cells of this mass are derived from CSCs and share common genetic elements with them. However, many CSCs elements, which may be important for diagnosis and prognosis, may be overlooked by researchers. This is especially true of epigenetic changes. One can predict that heterogeneity within the CSCs fraction must be higher than in the bulk, which consists mostly of differentiated and non-dividing tumor cells. A recent analysis of exomes [67] showed that the majority of somatic mutations in the CSCs fraction and in the bulk of the tumor are identical. These data can be interpreted as the result of constant dynamic transitions from CSCs to the differentiated state, and vice versa. This will be discussed below.

Many aspects of the CSC model remain controversial, but several experiments employing modern technologies to trace cells during development, including lineage-tracing studies, provide strong evidence in favor of a more or less stable existence of CSCs and a hierarchical organization of the tumor at least in some cases. These experiments were conducted in murine models of brain tumors [68], small intestine [69] and skin cancers [70], human colorectal adenomas [71], and gliomas [72] (see also comment in [73]). These studies showed that the majority of the investigated tumor cells had limited proliferative capacity and, apparently, originated from subpopulations with properties similar to those of CSCs.

## CSCS NICHES: DEFINITIONS AND CHALLENGES

It has been established that normal stem cells have so-called niches; physiological microenvironments composed of specialized cells that are involved in the regulation of stem cells, functioning through various types of signaling. In this case, the definition of a niche in which they exist is fairly straightforward [44, 45, 74-77]. It is a quite well-defined area around discreetly localized functioning stem cells in the tissue, although the existence of stem cells and their respective niches have not been demonstrated for all normal tissues [39]. The classic definition states that “stem-cell populations are established in ‘niches’ — specific anatomic locations that regulate how they participate in tissue generation, maintenance and repair. The niche saves stem cells from depletion, while protecting the host from over-exuberant stem-cell proliferation. It constitutes a basic unit of tissue physiology.” [78].

Using adult stem cells as an example, it has been proposed that CSCs also have niches and that interaction of CSCs with niches may regulate self-renewal, proliferation, and differentiation of CSCs [77, 79, 80]. However, in the case of CSC there are more questions than answers (see recent reviews [1, 81, 82]). Many authors (e.g., [79]) consider a solid tumor to be an “organ” which consists of cancer cells and the stroma, which occupies most of the tumor volume and creates a microenvironment that can be considered an analogue of normal cell niches.

The CSCs niche is a microenvironment which has no morphological structure [83]. However, according to some authors (see, e.g., review [84]), the CSCs niches differ from the overall microenvironment. The cells inside the niche produce factors that stimulate CSCs self-renewal, induce angiogenesis, and recruit immune and other stromal cells, which secrete additional factors that contribute to the invasion and metastasis of cancer cells.

The interaction of tumor stromal elements with putative CSCs is covered in a large number of publications. For example, there is evidence that anti-tumor agents affect putative CSCs *in vitro *and *in vivo* differently [43], and this may mean that some important components of CSCs regulation are provided by the microenvironment (niche). The microenvironment includes the extracellular matrix, mesenchymal and endothelial cells, immune system cells, adhesion molecules, various growth factors, and cytokines and their receptors [57, 85]. It is assumed that blood vessels can also play a role in the creation of niches [81], as they do in the case of normal stem cells. The tumor stroma secretes factors that regulate the behavior of cancer cells [80] and actively support the growth of the tumor via neo- and by angiogenesis [86]. The microenvironment determines the fate of a tumor, serves as a barrier for the therapeutic intervention, and can affect the plasticity of tumor cells, for example, transitions from CSCs to non-CSCs [8].

Two-way interactions between cancer cells and the stroma are widely discussed, especially, the role of stroma in tumor development and, in particular, the acquisition of such highly important qualities as invasiveness and metastatic potential [87]. It is believed that not only does the stromal niche affect cancer cells, but the reverse is also true: cancer cells (which mainly means CSCs) can affect the stroma as well and use it for their development [81], in particular, to create pre-metastatic niches [80, 85, 86, 88].

Despite the widely discussed importance of stromal niches in tumor development, there are very little data reliably confirming their function at the molecular level, as well as at the level of information transport; e.g., whether the transport is carried out in paracrine, autocrine, or in any other way [43].

More detailed information on this matter will help to better evaluate the real role of the niche in the development of tumors and to develop rational therapeutic strategies.

## NORMAL AND CANCEROUS NON-STEM CELLS CAN SPONTANEOUSLY TRANSFORM INTO A STATE SIMILAR TO STEM CELLS. APPARENTLY, THERE IS NO STRICT BARRIER BETWEEN CSCS AND NON-CSCS CANCER CELLS

Different types of cancers and, maybe, even the same type of cancer in different patients may follow either the CSCs model or the stochastic evolution model. In dealing with such labile systems as stem cells, cancer cells, or CSCs one should always consider the possibility of their phenotypic restructuring as a result of epigenetic processes. In 2011, three groups [89, 90] (see reviews [35, 91]) of researchers used cancer cell lines and primary tumors to describe the acquisition of a self-renewing capacity in non-CSCs populations. For example, they described mammary epithelial cells capable of spontaneous de-differentiation into a “stem-like” state [92]. The malignant transformation enhanced this ability, making it possible for ordinary cancer cells to transform into a state similar to CSCs’ *in vitro *and *in vivo*. These data demonstrated the high plasticity of stem cells in general and CSCs in particular, as well as the ease of interconversion of non-stem cells into stem cells and vice versa, especially in malignant tumors.

These findings provide ground to believe that such reversibility and lack of rigid hierarchy in stem and non-stem cells may be commonplace, making cancer therapy, in particular therapy aimed at CSCs, more challenging.

The observed plasticity of cancer cells and the possibility of transition between stem and non-stem cells introduce additional complexity in the study of the role of cancer stem cells in carcinogenesis. This plasticity may depend on a number of factors, with signals from the microenvironment and intercellular interactions in the niches playing an important role [8]. These transitions most likely are stochastic epigenetic or genetic events influenced by the microenvironment of cancer cells and intercellular interactions in the niches [20]. For example, CSCs can be converted into a non-CSC, and* vice versa *[8], existing in a dynamic equilibrium [93].

Therefore, one of the important aspects of the CSCs concept, i.e. the presence of an irreversibly separated CSCs fraction in the tumor mass (see above), is untenable overall [88, 91]. Differentiated cancer cells and CSCs are in a constant state of mutual transformation [94]. Environmental factors, including growth factors, can cause transitions between the states. Moreover, the plasticity of CSCs may cause a transformation of epithelial cancerous cells into mesenchymal (and *vice versa*) [6, 41, 67, 95-98]. This phenotypic plasticity is caused by both mesenchymal-epithelial and epithelial- mesenchymal transitions and, apparently, by genetic, epigenetic, and intracellular and intercellular signaling programs [58]. It can be assumed that the existence of CSCs and their plasticity depend on both internal factors (genetic and epigenetic architecture of cancer cells) and on the microenvironment. Since both types of factors are highly specific for the type of tumor and each individual patient, it is easy to imagine a situation where in some cases CSCs are present, and the barrier between CSCs and non-CSCs is high enough to produce an isolated permanent CSCs fraction, while in other cases this barrier is low and there is a constant interconversion of CSCs and non-CSCs cells. Finally, in some types of cancers and in some patients CSCs groups are not produced and the tumor evolution proceeds by the stochastic clonal mechanism.

## CONCLUSION: CSCS AS THERAPEUTIC TARGETS

Numerous publications are dedicated to the potential use of CSCs for therapeutic purposes. This is not surprising.

The bulk of the tumor mass has a limited proliferative capacity, and CSCs, if they really are the driving force behind the development of the tumor, represent only a small part of it. As a result, therapeutic agents act mainly on the highly differentiated part of the tumor and their efficiency is low. Targeting the active CSCs minority would greatly increase the efficiency of the therapy. Subsequently, in recent years many authors (see e.g. [59]) have considered (and still do consider) CSCs as a rather promising therapeutic target. New strategies and approaches to therapy focused on CSCs are being developed very intensively. The literature devoted to the principles and methods developed in this area is very abundant and contradictory. Readers can get acquainted with the proposed ideas and methods in a number of reviews published in 2015 [8, 59, 99-105]. CSCs features, which were targeted by therapeutic interventions, are listed in *Table 1*.

Despite the multitude of proposed approaches, there are only two basic strategies, which differ in the choice of target (see [35] and reviews, cited above). The first strategy targets surface antigens, which are presumably characteristic of CSCs. The second strategy relies on the fact that self-renewing putative CSCs are in many respects similar to embryonic stem cells (see, e.g. [35, 59]) and should express embryonic signaling pathways, which are not typical of adult cells [59].

Speaking of surface antigens, there are known cases of CSCs, normal cells, and normal stem cells having the same antigens. As a result, a therapy targeting surface antigens often causes serious side effects. For example, the therapeutic monoclonal antibodies gemtuzumab ozogamicin (GO, anti-CD33) and alemtuzumab (anti- CD52) have been recently withdrawn from the oncological market because of their toxicity. Therefore, the search for surface CSCs markers with higher selectivity continues [59].

Various components of the signaling pathways known to be involved in embryogenesis, such as Notch, Hedgehog (HH) and Wnt have been also investigated as targets for influencing CSCs. Some of them are listed in *Table 1* (see [59, 99] and others cited in reviews). It is still too early to judge the success of any particular therapy, because the trials are in their early stages (*Table 2*). However, it should be noted that treatment targeting embryonic signaling systems that are active in adult stem cells can cause serious side effects.

**Table 2:** Signaling pathways targeted in clinical trials for the inhibition of various cancers (modified from [105])

Cancer type	Targeted signaling pathway	Therapy: mono- or combination
Colon cancer	STAT/β-β-catenin /Nanog	Combination
Stomach cancer	STAT/β-β-catenin /Nanog	Monotherapy Combination
GI cancer	STAT/β-β-catenin /Nanog	Combination
Hepatocellular carcinoma	STAT/β-β-catenin /Nanog Wnt/FZD8Fc/FAK	Combination Combination
Mesothelioma	FAK	Monotherapy
Squamous cell lung cancer	Notch FAK	Combination Monotherapy
Testicular cancer	Notch FAK	Combination Combination
Pancreas cancer	STAT/β-β-catenin /Nanog Notch	Combination Combination
Small cell lung cancer	Notch	Combination

An article published in *Science *in early 2015 [102] covers various points of view on the existence of cancer stem cells and their significance for the evolution and treatment of cancer. In particular, it lists both practical and theoretical considerations on the feasibility of the use of cancer stem cells as the only therapeutic target.

In our review, we have attempted to address these issues from different perspectives. We believe that these data allow us to express doubt that a therapy aimed at CSCs will be successful. For example, it seems that easy transition between CSC and undifferentiated cells, and *vice versa*, will make it impossible to completely eradicate CSCs. Moreover, there are concerns about the potential emergence of cells that will initiate a new tumor from the remaining non-differentiated cancer cells. Clinical trials of drugs that target CSCs are in too early a stage to pass judgment on their success. Kaiser J. [102] in the conclusion of his article writes: “For now, cancer patients, researchers and physicians, and investors [of companies using CSCs as targets] … will anxiously wait for data to roll in from the clinical trials. For those with a stake in treatments, the results could bring hope. For researchers debating the reality of cancer stem cells, though, they may not bring resolution. Says Jeremy Rich of the Cleveland Clinic in Ohio, who is studying stem cells in brain cancer, “Even if we’re wildly successful, which I don’t think we will be, I don’t think there will be a black-and-white answer.”

In 1209 during the siege of Beziers, which was defended by true Catholics and heretics, Arnold Amalric, the papal legate and a prominent participant of the Albigensian Crusade, allegedly answered a question on how to distinguish true believers from heretics with “Kill them all and let God sort them out.” This cannot be applied to people, of course, but in our opinion it is the only right strategy for cancer therapy: there is no need to look for differences between cancer cells, just kill them all. In our work, we adhere to this principle, combining two strategies of total eradication of cancer cells: suicide gene therapy and immunotherapy. The results are very promising, at least in preclinical trials [106].

The analysis of clinical trials [105], examples of which are listed in *Table 2*, shows that the majority of researchers are also using a combination therapy, which is absolutely justified given the extreme plasticity of CSCs.

## EDITORIAL NOTE

Oncological diseases kill millions of people each year. Despite the significant progress made in recent years, cancer remains far from defeated. What’s more, that is unlikely to happen in the next ten years. The more we learn about the molecular and cellular basis of the malignant transformation, the more intractable the problems, whose solution is required for successful treatment, seem.

From the prevailing point of view, cancer is a set of diseases underlain by disturbances in the basic processes of cell metabolism. A baffling variety of these processes determines both the origin and course of the malignant transformation and the set of approaches to treatment. Investigation of these processes in the last decades has been littered with victories and defeats, and it is arduous to say which will prevail.

Today, there is a wide variety of sometimes opposing viewpoints on cancer. In our opinion, a discussion of this topic should be of interest to everyone. In this issue, we are opening the discussion with an article by T.V. Vinogradova, I.P. Chernov, G.S. Monastyrskaya, L.G. Kondratyeva, and E.D. Sverdlov “Cancer stem cells: plasticity versus therapy.” We emphasize once again the disputable nature of the publication, but it reflects the authors’ opinion. At the same time, we believe that this publication will give rise to further discussion of the issue.

**We are awaiting your reactions!**

## Abbreviations

CSCs: cancer stem cells
SPM: stemness phenotype model
